# Supplementary material for: HIVprotI: an integrated web based platform for prediction and design of HIV proteins inhibitors
Source: J Cheminform. 2018 Mar 9;10:12. doi: 10.1186/s13321-018-0266-y (PMC5845081; doi:10.1186/s13321-018-0266-y)
Supplement: Supplementary file 2 — Additional file 2. Source code of HIVProtI web server. [file 13321_2018_266_MOESM2_ESM.zip › HIVprotI_Source-code/hivprotI/weka-3-6-6/doc/weka/experiment/Task.html]

Task


---


|  |  |  |
| --- | --- | --- |
| **Overview** \| **Package** \| **Class** \| **Tree** \| **Deprecated** \| **Index** \| **Help** \| **Weka's home** | |  |
| **PREV CLASS**   **NEXT CLASS** | **FRAMES**    **NO FRAMES**     **All Classes** |  |
| SUMMARY: NESTED \| FIELD \| CONSTR \| METHOD | DETAIL: FIELD \| CONSTR \| METHOD |  |


---


## weka.experiment Interface Task

**All Superinterfaces:**: java.io.Serializable

**All Known Implementing Classes:**: RemoteBoundaryVisualizerSubTask, RemoteExperimentSubTask

---

``` public interface Task extends java.io.Serializable ```

Interface to something that can be remotely executed as a task.

**Version:**
:   $Revision: 1.8 $

**Author:**
:   Mark Hall (mhall@cs.waikato.ac.nz)

---

| **Method Summary** | |
| --- | --- |
| `void` | `execute()`             Execute this task. |
| `TaskStatusInfo` | `getTaskStatus()`             Clients should be able to call this method at any time to obtain information on a current task. |

**Method Detail**

### execute

```
void execute()
```

:   Execute this task.

---


### getTaskStatus

```
TaskStatusInfo getTaskStatus()
```

:   Clients should be able to call this method at any time to obtain
    information on a current task.

    :   **Returns:**: a TaskStatusInfo object holding info and result (if available) for this task


---


|  |  |  |
| --- | --- | --- |
| **Overview** \| **Package** \| **Class** \| **Tree** \| **Deprecated** \| **Index** \| **Help** \| **Weka's home** | |  |
| **PREV CLASS**   **NEXT CLASS** | **FRAMES**    **NO FRAMES**     **All Classes** |  |
| SUMMARY: NESTED \| FIELD \| CONSTR \| METHOD | DETAIL: FIELD \| CONSTR \| METHOD |  |


---
